# Modern Sociology

**Published:** 1900-12-15

**Authors:** 


### Modern Sociology.

### DANGEROUS TRADES.

### Dyeing and Dry Cleaning.

We all know that, as many dyes are composed of poisonous substances, special precautions for the protection of the workers must be taken in the use of them, but to comparatively few has it occurred that the process known as "dry cleaning" has any dangers attached to it.

The term "dry cleaning" is, strictly speaking, a misnomer. The soiled goods are cleaned with a liquid, benzine or naphtha, but this evaporates so quickly that the goods are, practically speaking, dry as soon as they are clean. When dry cleaning was merely a domestic industry—a matter of scrubbing with benzine and a brush—the dangers were very great, for it was often done in kitchens, with cooking going on at the same time, or in rooms placed high up where there was no chance of escape in case of fire. Yet the substances used in the process of cleaning are in the highest degree inflammable. Nowadays, when dry cleaning has been extended to larger garments, the work is generally done in factories, where greater precautions are taken, though the standard of protection is not equally high in all, and, even in the best, fires occasionally take place. As the fires are small and are generally put out before any accident can take place, their occurrence does not often come to the public ear.

The precautions taken are, first and chief, the careful looking over and searching of all goods sent to be cleaned. A match left in a pocket or the lining of a garment may occasion a fire, or one may even originate spontaneously in the process of cleaning. Thus, on a thundery day, the rubbing of a silk fabric with benzine may generate an electric spark with disastrous results. The spontaneous combustion of benzine may, however, be avoided by the addition of a small quantity of oil soap. The best firms are very careful in these matters, but others, anxious to economise and perhaps undersell their neighbours, are neglectful, and, as often happens, the workers themselves seem to be very careless, as the following incident will show:—A valuable carpet had been sent to a certain factory to be cleaned. It was treated and rinsed just before closing-time, and after it had been hanging up for half an hour or so in the factory, the foreman, knowing it to be valuable, took it home to dry, and hung it up in front of the fire where his two children were playing. In about ten minutes the heat of the fire caused the carpet to take fire, and the children were so severely burnt that they died within twelve hours. Under such a foreman as this, can it be wondered at that fires take place in dry-cleaning factories? Perhaps a preliminary examination in chemistry ought to be exacted from all seeking posts of trust in such places.

Besides this serious danger there is a more constant one. In speaking of inflammable paints we noted that those who worked with them are subject to nausea, giddiness, and occasional intoxication and lack of consciousness. The same symptoms, springing from the same cause—the evaporation of the spirit—are to be found among the workers in cleaning factories. In some of the lighter departments of the work girls are employed, and several of these told the committee that "the spirit got into their heads and made them act silly." All the girls admitted that they "tasted the spirit in their food," and some of them suffered from headache. The sufferers from headache felt the intoxicating effect more when their heads ached, and felt more than usually tired in the evenings.

To prevent these dangers the committee recommend that the tanks or vessels in which the goods are swirled round to be cleaned or rinsed should have a closed but not fastened lid, which would, if it was lifted up by the occurrence of an explosion in the vessel, such as cannot but happen sometimes, be heavy enough to fall again by its own weight, and by keeping out the air extinguish the fire. They recommend also the keeping of sand in all places where benzine or naphtha is used, and having blankets about in case of fire. To this end also they advise that the workers should wear woollen clothing. They think that where cleaning is carried on in a room above the ground floor an outside emergency staircase should be provided. An abundant water supply and the means of using it, should be at hand, and, if possible, electric light should be used. All young persons and women employed should be examined once a month by a medical man, who should have power to order total or temporary suspension of work.

The dangers attaching to the use of many dyes has already been recognised by the legislature, but lately attention has been drawn to the risk of lead poisoning springing from the use of acetate or brown sugar of lead in the dyes for producing the chrome colours—viz., orange, yellow, lemon, and green. It is with regard to orange that the danger is strongest, for all the others are weaker solutions of the dye, supplemented, in the case of green, by a bath of indigo. To obtain the deep orange colour, hanks of yarn are first dipped into a solution of lime and then into brown sugar of lead solution; they are then passed again through a lime solution and a lead solution, after which they are dipped into a vat containing bichromate of potash, and finally boiled in lime water. In the lighter colours the second lead bath is omitted. In the mere dyeing of the yarn or cloth, there is little danger to the health of those employed, but when the yarn has to be dried and bundled, or "noddled," as it is called in the trade, an amount of dust, either of the chromate of lead itself or of particles of the fabric coated with the chemical, is set free which those who are handling the yarn can hardly help inhaling. In calico printing a similar, though less serious danger occurs. There the pattern is first printed in lead salts, after which the goods are passed through a solution of bichromate of potash or soda. Only the parts which have been coated with the lead solution retain the chrome and come out coloured. The calico then passes on an endless blanket into a drying stove, and it is here, rather than in the dyeing, that risks occur. Boys are at work here to see that the work does not crumple, but is evenly folded, and also to turn or change the blanket when necessary. With the drying, particles of dust charged with lead are liberated, and there is again the risk of the workers being affected with lead poisoning. Sometimes a perfect epidemic of lead poisoning has resulted in calico factories from the diffusion of lead in drying either the yarn or the printed cloth. When fans were put up, and special care taken of the health of the workers, the disease decreased. But even yet the regulations for the protection of workers in these factories are insufficient. Thus it is ordained that towels and basins should be provided for the use of workers, but no regulations exist as to the proportion between the number of conveniences, and that of those who are to use them. The committee recommend that a basin, &c., should be provided in the proportion of one to every five workers. This is not excessive. It is, indeed, far below the allowance in some American workshops, but it would be a great improvement on many here. They recommend also that young persons be not allowed to undertake any of these poisonous processes, and that all workers should wear overalls. They also advise that the employer should insist on workers washing their hands and face before meals, but this, it may be feared, is beyond even employers' power. More within their domain is the recommendation to put hoods or cowls close to the bar where the yarn is noddled, with a fan to draw the dust away from the face of the worker.

